# 3-dimensional intracardiac echocardiography for structural heart interventions

**DOI:** 10.3389/fcvm.2023.1180299

**Published:** 2023-11-07

**Authors:** Edwin C. Ho, Manaf Assafin, Tadahisa Sugiura, Juan F. Granada, Mei Chau, Azeem Latib

**Affiliations:** ^1^ Division of Cardiology, Montefiore Medical Center, Albert Einstein College of Medicine, New York, NY, United States; ^2^ Division of Cardiothoracic Surgery, Montefiore Medical Center, Albert Einstein College of Medicine, New York, NY, United States; ^3^ Cardiovascular Research Foundation, New York, NY, United States

**Keywords:** intracardiac echocardiography, 3-dimensional intracardiac echocardiography, transcatheter mitral intervention, transcatheter tricuspid intervention, interventional echocardiography, structural heart intervention, atrial septal defect closure, paravalvular regurgitation closure

## Abstract

New generation 3-dimensional matrix array intracardiac echocardiography catheters have become commercially available recently, increasing image plane options compared to 2-dimensional and older generation 3-dimensional intracardiac echocardiography catheters. They are beginning to play an important role in structural heart interventions, especially for transcatheter tricuspid interventions, due to advantages in some situations that increase image quality over transesophageal echocardiography.

## Introduction

Transcatheter structural heart interventions rely heavily on live echocardiographic guidance performed by interventional imaging specialists to achieve safe, efficient and effective procedures (1). The most commonly used echocardiography imaging modality during procedures is transesophageal echocardiography (TEE), since it has the ability to provide detailed, high quality 2-dimensional and 3-dimensional visualization of all cardiac structures (2).

New generation 3-dimensional matrix array intracardiac echocardiography (ICE) catheters have become commercially available in the United States serving as an important adjunctive imaging modality to TEE in structural heart interventions. ICE has several advantages over TEE, specifically offering near field imaging and avoiding intracardiac acoustic shadowing in some situations. There are also reduced sedation requirements, potentially allowing certain procedures to be performed without general anesthesia and mechanical ventilation.

## Commercially available 3-dimensional ICE platforms

Three 3-dimensional ICE platforms are already commercially available in the United States. All three technologies are capable of single plane 2-dimensional imaging, biplane imaging, live 3-dimensional imaging and live 3-dimensional multi-planar reconstruction.

The Siemens AcuNav Volume (Siemens Healtineers, Erlangen, Germany) has been available for a longer period of time than the newer matrix array probes. The AcuNav Volume is a 12.5 French system with a 128-element helical transducer element array. It can provide a live 3-dimensional volume size up to 90 × 50 degrees. The Siemens ACUSON SC2000 echocardiography console is used to generate and control 2- and 3-dimensional images. Four degrees of flexion including anterior, posterior, left and right are possible.

The Philips VeriSight Pro (Philips Healthcare, Andover, MA, USA) catheter is a 9 French system with an 840-element matrix array. The maximum 3-dimensional volume size is 90 × 90 degrees. It is compatible with the Philips Epiq CVxi, CVx and 7C consoles. Anterior, posterior, left and right flexion are controlled using two rotary knobs on the probe handle.

The Biosense Webster NuVision (Biosense Webster, Irvine, CA, USA) catheter is a 10 French system with an 840-element matrix array. The maximum 3-dimensional volume size is also 90 × 90 degrees. It is compatible with the GE E95 and S70N (GE Healthcare, Chicago, IL, USA) consoles. Uniquely, while there is only anterior and posterior flexion, the probe has the capability of rotating the transducer array 360 degrees around the long axis of the catheter independently of the catheter itself.

## 3-dimensional ICE compared to 3-dimensional TEE

It is important to recognize that 3-dimensional ICE is not identical to 3-dimensional TEE and important differences exist. 3-dimensional TEE probes have much larger matrix arrays (such as the 2,500 elements found on a Philips X8t TEE probe), which provide better spatial and temporal resolution. The maximum 3-dimensional volume size is also larger on TEE. Biplane image quality is more consistent across all angles relative to the transducer array on TEE, vs. ICE which suffers spatial resolution degradation perpendicular to the long axis of the catheter (at 90 degrees on the omniplane angle) due to physical transducer array limitations. As a result, optimal biplane imaging on 3-dimensional ICE is achieved when the imaging planes are closer to diagonal across the matrix array, such as at 45 and 135 (or −45 and +45) degrees. Lastly, while TEE probes are multi-use, 3-dimensional ICE catheters are single use.

With respect to the omniplane angle, 0 degrees on an ICE probe corresponds to the imaging plane that is parallel to the long axis of the catheter, whereas 0 degrees on a TEE probe corresponds to the imaging plane that is perpendicular to the long axis of the probe.

## Workflow considerations

One of the natural questions that is raised by the introduction of 3-dimensional ICE into the transcatheter intervention space is how the imaging expert fits into the workflow of this imaging modality. While some may argue that these catheters open the possibility of minimizing personnel and removing the interventional imager from the room, there is a considerable amount of expertise in image optimization, biplane and multi-planar reconstruction generation, image interpretation, and procedural decision making that an expert imager can contribute. Additionally, fusion imaging systems are expected to incorporate 3-dimensional ICE soon, and in some systems, control of the fusion imaging platform is located on the echocardiography machine.

Unlike TEE, direct manipulation of the imaging transducer and console control are generally physically separated in space and by the sterile field. One workflow option can have the interventional imager performing catheter manipulation to optimize the transducer position and imaging plane, especially since the same physics principles considered during TEE probe manipulation apply. Unfortunately, current platforms do not allow full console manipulation from the sterile field. Therefore, the interventional imager's skills may be better used at the echo machine rather than at the probe, since 3-dimensional ICE allows for enough digital imaging plane manipulation options to minimize the need for physical probe position changes.

## 3-dimensional ICE for transcatheter mitral interventions

**Figure 1:**
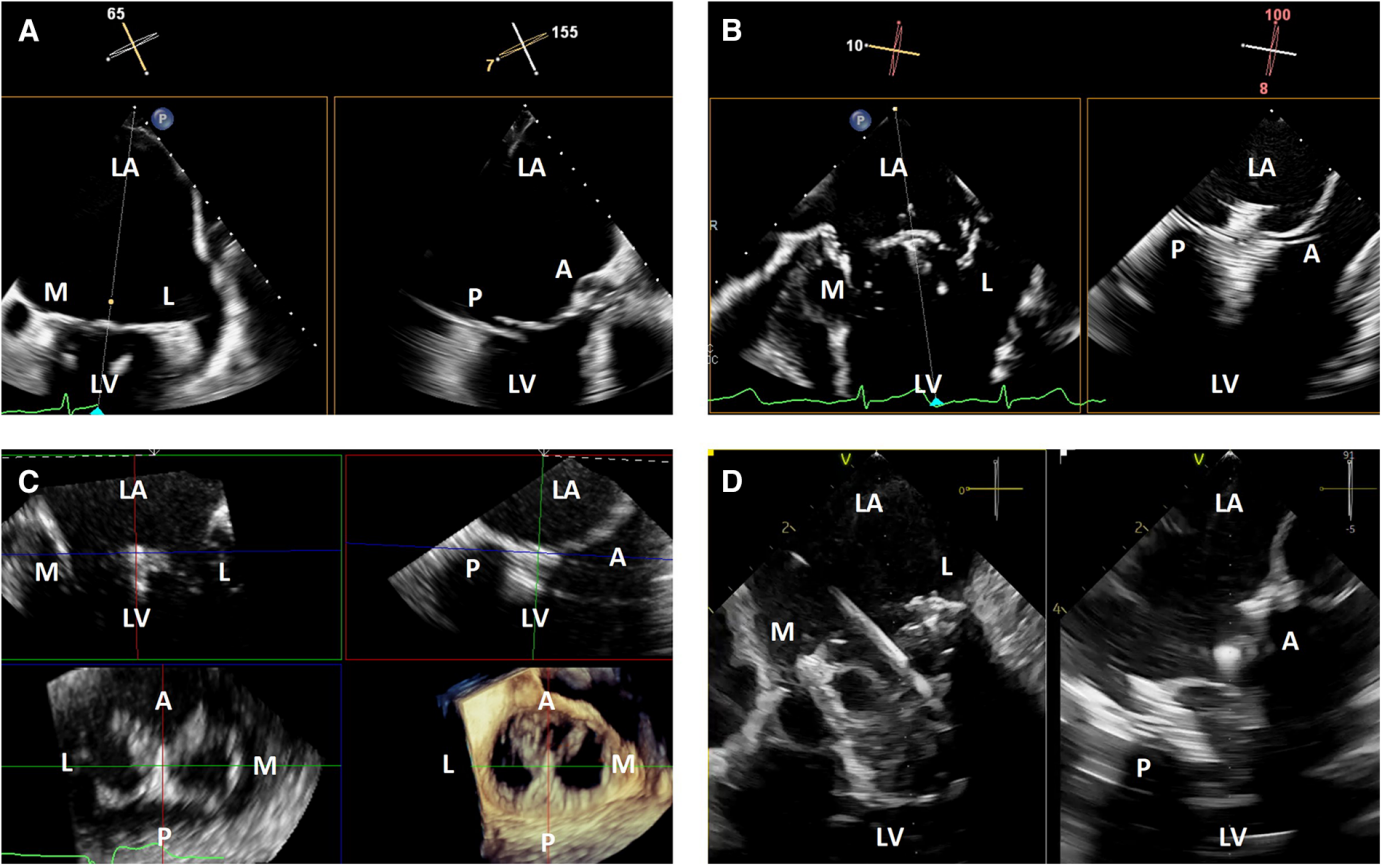
3-dimensional intracardiac echocardiography for transcatheter mitral interventions. (**A**) Biplane transesophageal echocardiography in a severely dilated left atrium causing reduced spatial resolution of the mitral valve due to its far field position; (**B**) biplane intracardiac echocardiography in the same patient with improved visualization of the mitral valve, allowing for a successful mitral transcatheter edge-to-edge repair; (**C**) live 3-dimensional multi-planar reconstruction from an intracardiac echocardiography volume after deployment of a mitral transcatheter edge-to-edge repair device; (**D**) biplane intracardiac echocardiography during a transcatheter mitral valve-in-valve implantation. LA, left atrium; M, medial; L, lateral; LV, left ventricle; A, anterior; P, posterior.

TEE imaging is excellent for transcatheter mitral interventions due to the position of the esophagus relative to the left atrium. The mitral valve is typically less than 10 cm from the probe, and when imaged from the left atrial wall opposite from the mitral valve, is well-positioned for flexible imaging of the entire valve using single plane, biplane or 3-dimensional imaging. When the left atrium is massively enlarged, the esophagus is significantly displaced laterally, or when other anatomic abnormalities limit TEE image quality, ICE may provide better intraprocedural imaging (Figure 1).

Optimal visualization of the mitral valve is generally achieved by positioning the ICE probe in the left atrium across a trans-septal puncture, by first positioning the tip of the ICE probe near the puncture site and then crossing using anterior flexion and gentle catheter advancement. This is usually done through the same puncture site as the device delivery system, with the main advantages being reduced trauma to the interatrial septum and increased simplicity compared to requiring a second separate puncture. The disadvantages include possible interaction between the delivery system and ICE catheter during delivery system manipulation, which may cause undesired movement of the ICE probe. The close proximity between the ICE probe and delivery system may also cause increased acoustic shadowing if the ICE probe is positioned in such a way that the catheters are in the line of sight between the transducer array and mitral valve. Using two separate punctures may reduce interaction and shadowing from the delivery system, at the cost of slightly increased procedural complexity and increased trauma to the interatrial septum.

**Figure 2:**
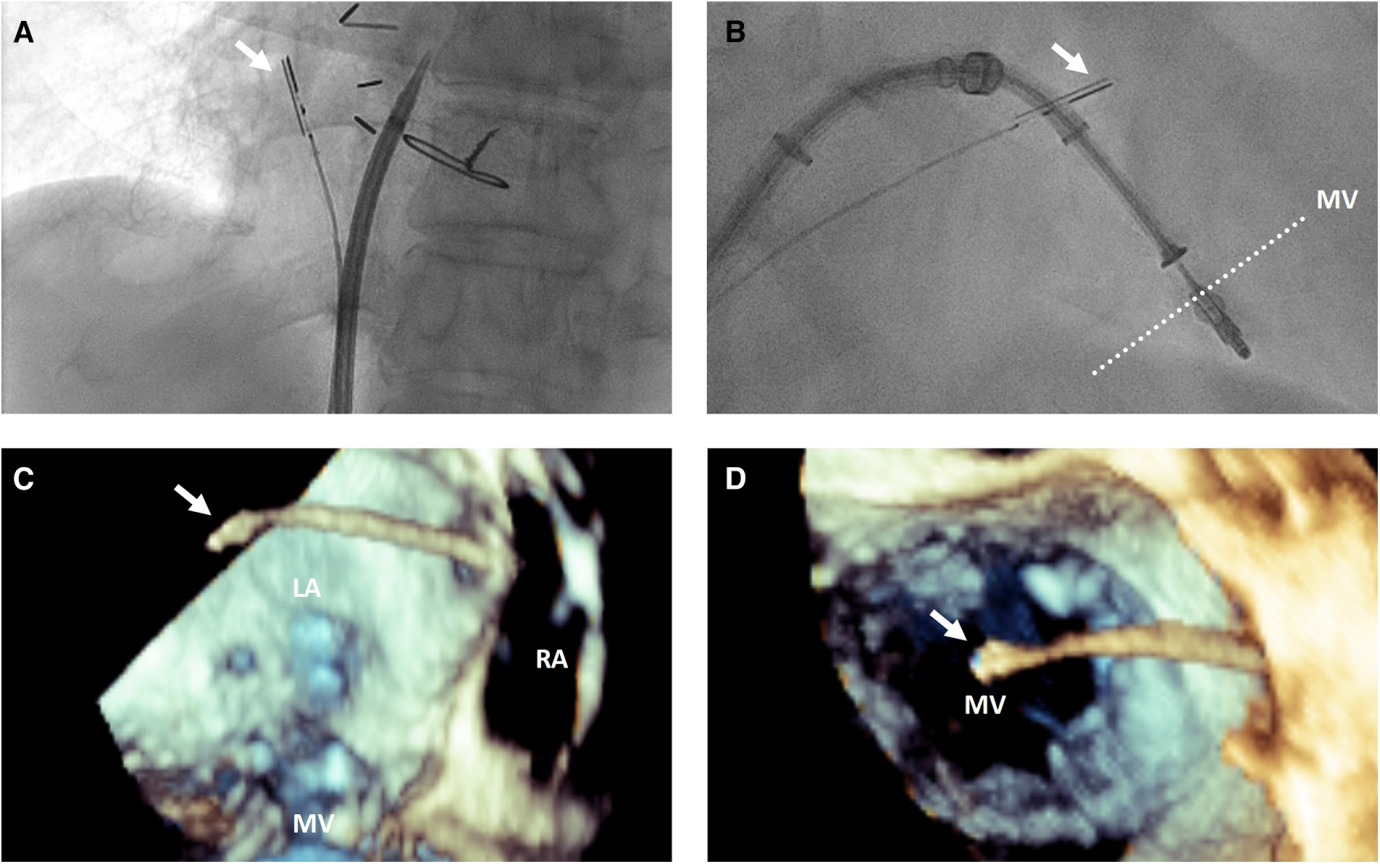
3-dimensional intracardiac echocardiography probe positioning for transcatheter mitral interventions. (**A**) Fluoroscopic view demonstrating the intracardiac echocardiography probe (white arrow) positioning with posterior flexion relative to a trans-septal puncture system to image the interatrial septum for trans-septal puncture; (**B**) fluoroscopic and 3-dimensional transesophageal echocardiographic views from posterior looking anterior (**C**) and from a surgeon's view above the mitral valve from the left atrium (**D**) demonstrating the intracardiac echocardiography probe (white arrow) positioning relative to the mitral valve for intraprocedural guidance of a transcatheter edge-to-edge repair. LA, left atrium; RA, right atrium; MV, mitral valve.

Once the ICE probe is positioned in the left atrium, the face of the imaging array should be positioned as parallel to the mitral annulus as possible using the appropriate amount of anterior or posterior flexion, although additional adjustments may be needed to avoid or minimize acoustic shadowing from delivery system catheters (Figure 2). Left or right flexion can then be used to optimize the anterior to posterior position within the left atrium, with the goal to place the imaging array as centrally over the mitral valve as possible.

Angulation of the asymmetric imaging array relative to the mitral coaptation line is also affected by left or right flexion. To maximize biplane quality for leaflet segment coaptation pair visualization, such as for leaflet grasping in transcatheter edge-to-edge repair, it is best to obtain an intercommissural view at either −45 or +45 degrees so the orthogonal “grasp” or long axis view is taken at +45 or +135 degrees. As described above, this is to overcome the image degradation that occurs perpendicular to the long axis of the ICE probe (at 90 degrees) due to the crystal arrangement of the ICE matrix array. After optimization, good quality biplane, live 3-dimensional and live 3-dimensional multi-planar reconstruction can be performed similarly to TEE imaging (Figure 1).

Another window where the mitral valve and subvalvular apparatus are well-visualized is from the right ventricle. After crossing the tricuspid valve, rotation of the catheter to face the left ventricle can provide long and short axis views of the mitral valve from the ventricular perspective.

Steering devices through the left atrium may require a different ICE position to better visualize the left atrial free wall, left atrial appendage and pulmonary veins. This can be achieved by posterior flexion of the probe in the left atrium so it is resting almost against the interatrial septum.

Other special situations where 3-dimensional ICE can be considered for mitral interventions are in situations where TEE is extremely technically difficult, contraindicated due to anatomical factors (such as esophageal pathology), or the risk of general anesthesia and mechanical ventilation is very high or prohibitive (3, 4). In the future, certain procedures may be safely performed with minimal sedation to reduce procedural risk and hospital length of stay, but further data to support this hypothesis is needed (5).

## 3-dimensional ICE for transcatheter tricuspid interventions

**Figure 3:**
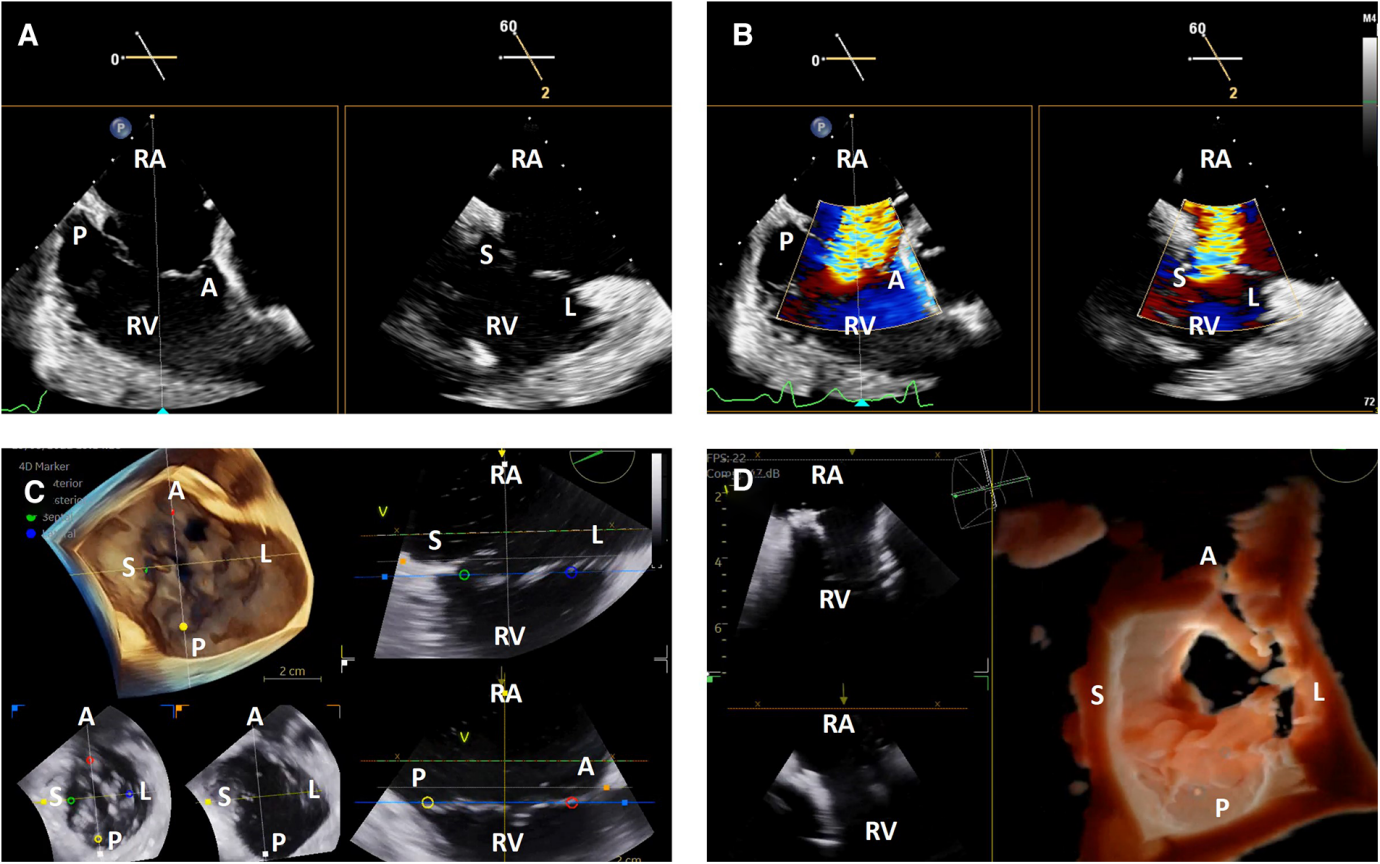
3-dimensional intracardiac echocardiography for transcatheter tricuspid interventions. Biplane intracardiac echocardiographic evaluation of the tricuspid valve without (**A**) and with (**B**) color Doppler; (**C**) live 3-dimensional multi-planar reconstruction of the tricuspid valve with color-coded anatomic markers and two independently controlled short axis views on the bottom left (leaflet tip level on the left, annulus level on the right); **(D**) 3-dimensional *en face* view of a degenerated prosthetic tricuspid valve in diastole with two simultaneous 2-dimensional image plane reconstructions, obtained during a tricuspid valve-in-valve implantation. RA, right atrium; RV, right ventricle; A, anterior; P, posterior; S, septal; L, lateral.

TEE image quality during transcatheter tricuspid interventions is variable due to the position of the tricuspid valve relative to the TEE probe. When the atria are dilated, the tricuspid valve may be in the far field for TEE. There may also be significant acoustic shadowing from intracardiac structures and prostheses. Lastly, cardiac chamber dilation often causes esophageal displacement relative to the atrioventricular valves, creating a “horizontal” appearance and further acoustic shadowing of the valve once delivery catheters are placed in the heart. Due to these factors, 3-dimensional ICE has significant advantages over TEE. The right atrial position of the probe places the tricuspid valve in the near field and avoids acoustic shadowing from left-sided structures and the crux of the heart (Figure 3).

The tricuspid valve is well-visualized using ICE without significant manipulation. Once the catheter is introduced into the right atrium from the inferior vena cava, the tricuspid valve is immediately visualized in the ICE “home view”. From this position, left or right flexion can be used to optimize angulation of the transducer face relative to the commissures to obtain the best biplane and 3-dimenional imaging, similar to what is described above for the mitral valve (6).

One of the critical TEE views used in transcatheter tricuspid interventions is the *en face* view where all leaflets are visualized in short axis (7). This is typically obtained from the shallow transgastric TEE window. 3-dimensional ICE catheters in the right atrium are not positioned to replicate this view with 2-dimensional imaging, and while live 3-dimensional imaging can provide this view, it does not have the same spatial and temporal resolution as TEE. As a result, the lack of this view may be a limitation for some transcatheter tricuspid interventions and current 3-dimensional ICE workflows often combine ICE with TEE imaging (Figure 3).

## 3-dimensional ICE for transcatheter atrial septal interventions and trans-septal puncture

The interatrial septum is well visualized by ICE after clockwise rotation of the probe from the “home view” to face the interatrial septum. Posterior flexion is helpful to position the probe away from contacting the septum directly to better visualize the fossa (Figure 2). A small degree of left or right flexion may also help to bring the superior vena cava in view to achieve a bicaval-like view. A greater degree of left or right flexion may bring the aortic valve in view to achieve an aortic short axis view.

**Figure 4:**
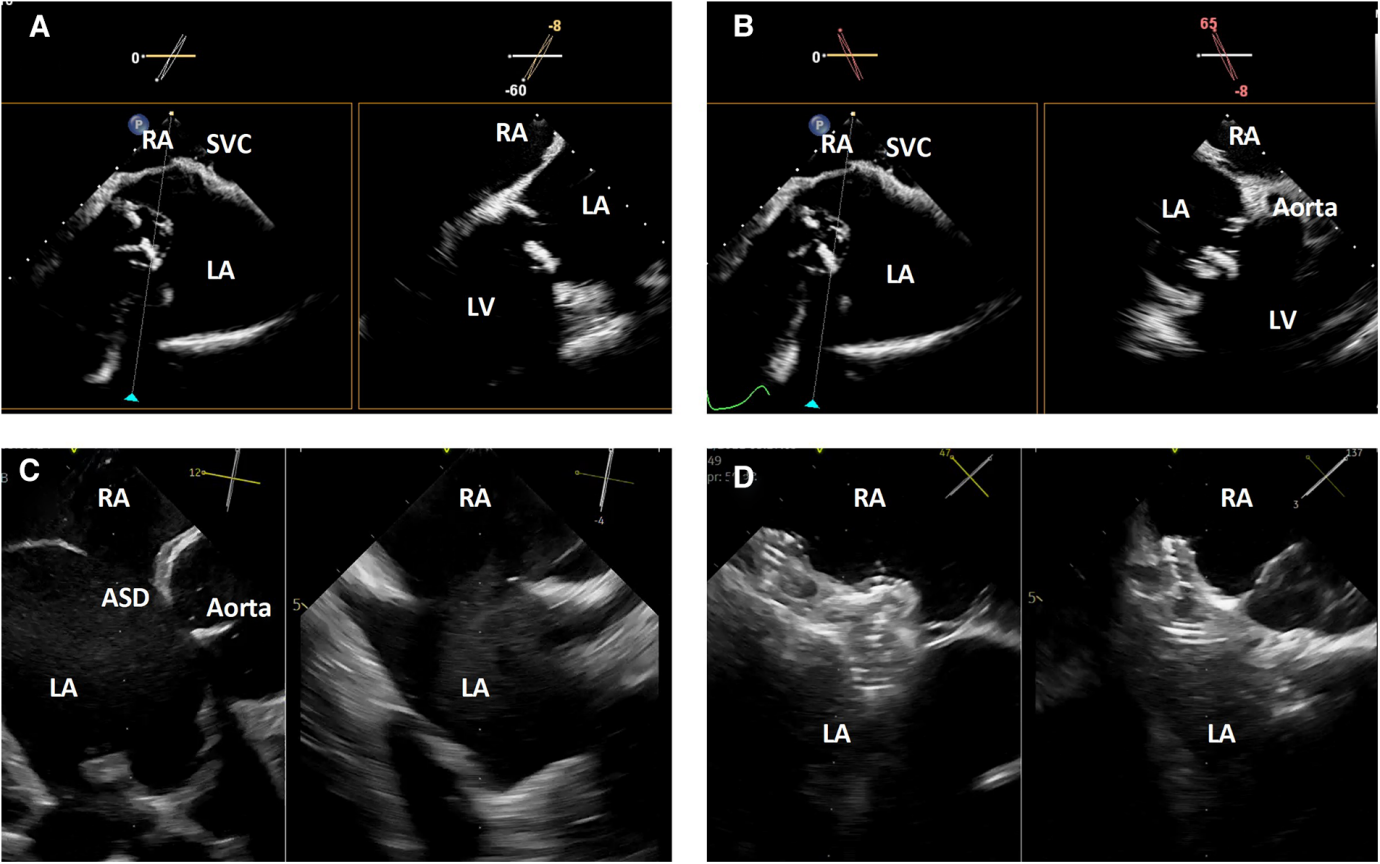
3-dimensional intracardiac echocardiography for transcatheter atrial septal evaluation and interventions. (**A**) Biplane intracardiac echocardiography evaluation of the interatrial septum in bicaval-like (left) and 4-chamber-like (right) views; (**B**) biplane intracardiac echocardiography evaluation of the interatrial septum in bicaval-like (left) and aortic short axis (right) views; (**C**) biplane intracardiac echocardiography evaluation of an atrial septal defect in aortic short axis (left) and orthogonal (right) views; (**D**) biplane intracardiac echocardiographic guidance of a transcatheter atrial septal defect closure. RA, right atrium, SVC, superior vena cava; LA, left atrium; LV, left ventricle; ASD, atrial septal defect.

Once the catheter is positioned, biplane or live 3-dimensional multi-planar reconstruction can be used to evaluate the interatrial septum by reproducing planes similar to those used with TEE image guidance of trans-septal punctures or atrial septal interventions. This includes a bicaval-like view, aortic short axis view and 4-chamber view (Figure 4).

In some atrial septal interventions, such as patent foramen ovale closure or simpler atrial septal defect closures, the use of ICE image guidance allows these procedures to be performed without general anesthesia and may reduce procedure-related costs, risks, and length of stay (8, 9).

## 3-dimensional ICE for left atrial appendage closure

ICE is being used more and more for left atrial appendage closure and 3-dimensional ICE has some potential advantages. In addition to biplane imaging to evaluate device positioning, 3-dimensional multi-planar reconstruction allows for appendage sizing similar to TEE. Precise trans-septal puncture can be performed using the techniques described above.

While the left atrial appendage is well visualized across the interatrial septum from the right atrium, image quality can be improved by crossing into the left atrium across a trans-septal puncture. Positioning the transducer to face the left atrial appendage by using posterior flexion to place it almost on the interatrial septum allows for optimal 2-dimensional, biplane and 3-dimensional image quality (10).

## 3-dimensional ICE for paravalvular leak closure

In addition to excellent visualization of the mitral and tricuspid valves as described above, aortic valve imaging planes can also be achieved using 3-dimensional ICE. Clockwise rotation from the home view and adjustment of anterior-posterior flexion such that the probe sits near the tricuspid annulus inferior to the aorta combined with slight leftward flexion can often visualize the aortic valve in short axis plane clearly. Additional short axis and long axis views can also be achieved from within the right ventricle. As a result, closure of mitral, tricuspid and aortic prosthetic paravalvular leaks can be guided by 3-dimensional ICE.

3-dimensional ICE guided paravalvular leak closure may have the unique advantage of closer probe proximity to the prosthesis and defect, which may visualize the leak, catheters and wires even more clearly than by TEE. This may be particularly true for aortic paravalvular leaks, which can be challenging to visualize and guide by TEE due to the distance of the aortic valve from the probe and significant acoustic shadowing due to the prosthesis itself. 3-dimensional ICE guidance may also permit the avoidance of general anesthesia to reduce procedural risks and length of hospital stay.

## Limitations

There are definite limitations to the current widespread adoption and successful use of 3-dimensional ICE for transcatheter structural interventions. Catheter cost is one of the primary barriers, but imaging platform compatibility and appropriate reimbursement, especially for interventional imaging experts, are also important factors in most centers. The inability to fully replicate all TEE views and some image quality limitations, as described above, are potential procedural limitations. Expertise, training, and workflow optimization are also important factors that will need to be addressed.

## Conclusion

3-dimensional ICE has a promising role in transcatheter structural heart interventions, especially when TEE imaging is technically challenging, or minimization of sedation requirements is desired. The overall experience of the structural heart community is growing, but ongoing work is needed to refine techniques and workflow to maximize the utility of this imaging modality.
